# Survivin drives tumor-associated macrophage reprogramming: a novel mechanism with potential impact for obesity

**DOI:** 10.1007/s13402-021-00597-x

**Published:** 2021-03-12

**Authors:** E. Benaiges, V. Ceperuelo-Mallafré, A. Madeira, R. Bosch, C. Núñez-Roa, M. Ejarque, E. Maymó-Masip, I. Huber-Ruano, M. Lejeune, J. Vendrell, S. Fernández-Veledo

**Affiliations:** 1 Institut d’Investigació Sanitària Pere Virgili, Endocrinology and Nutrition Service, Hospital Universitari de Tarragona Joan XXIII, 43007 Tarragona, Spain; 2 CIBER de Diabetes y Enfermedades Metabólicas Asociadas (CIBERDEM)-Instituto de Salud Carlos III (ISCIII), 28029 Madrid, Spain; 3 Rovira i Virgili University, 43003 Tarragona, Spain; 4 Grup de Recerca en Patologia Oncològica i Bioinformàtica, Molecular Biology and Research Section, Hospital de Tortosa Verge de la Cinta, IISPV, URV, 43500 Tortosa, Spain

**Keywords:** Survivin, Obesity, Cancer, Tumor-associated macrophages, Adipose-derived stem cells

## Abstract

**Purpose:**

Recent studies point to adipose-derived stem cells (ASCs) as a link between obesity and cancer. We aimed to determine whether survivin, which is highly secreted by ASCs from subjects with obesity, might drive a pro-tumoral phenotype in macrophages.

**Methods:**

The effect of ASC conditioned medium on the macrophage phenotype was assessed by expression studies. Survivin intracellular localization and internalization were examined by subcellular fractionation and immunofluorescence, respectively. Loss- and gain-of-function studies were performed using adenoviral vectors, and gene expression patterns, migration and invasion capacities of cancer cells were examined. Heterotypic cultures of ASCs, macrophages and cancer cells were established to mimic the tumor microenvironment. Survivin-blocking experiments were used to determine the impact of survivin on both macrophages and cancer cells. Immunohistochemical analysis of survivin was performed in macrophages from ascitic fluids of cancer patients and healthy controls.

**Results:**

We found that obese-derived ASCs induced a phenotypic switch in macrophages characterized by the expression of both pro- and anti-inflammatory markers. Macrophages were found to internalize extracellular survivin, generating hybrid macrophages with a tumor-associated phenotype that included secretion of survivin. Exogenous expression of survivin in macrophages generated a similar phenotype and enhanced the malignant characteristics of cancer cells by a mechanism dependent on survivin phosphorylation at threonine 34. Survivin secreted by both ASCs from subjects with obesity and tumor-associated macrophages synergistically boosted the malignancy of cancer cells. Importantly, survivin was mainly detected in ascites-associated macrophages from patients with a malignant diagnosis.

**Conclusion:**

Our data indicate that survivin may serve as a molecular link between obesity and cancer and as a novel marker for tumor-associated macrophages.

**Supplementary Information:**

The online version contains supplementary material available at 10.1007/s13402-021-00597-x.

## Introduction

Abdominal obesity, specifically an increase in visceral adipose tissue (VAT) mass, is an established risk and progression factor for many cancers [1]. Signatures of the insulin-resistant state, including obesity-related chronic low-grade inflammation and aberrant production of adipokines (leptin, adiponectin, visfatin, and plasminogen activator inhibitor-1) and proinflammatory cytokines (interleukin [IL]-6, IL-8, monocyte chemoattractant protein-1 [MCP-1] and tumor necrosis factor-α (TNF-α), are established drivers of cancer development [2–4]. In this context, there is considerable interest in the role of adipose-derived stem cells (ASCs) in tumor development. ASCs are progenitor cells of mesenchymal origin that reside within the stromal vascular fraction of adipose tissue [5, 6]. Remarkably, ASCs have been reported to travel through the blood to distant tumor sites, where they differentiate into vascular pericytes and secrete growth factors that support the tumor microenvironment [7–9]. Accordingly, ASCs are believed to play a central role in tumor growth, development and aggressiveness [10–12].

In addition to tumor cells, the tumor microenvironment contains a complex network of immune cells, adipocytes, myofibroblasts and mesenchymal stem cells, including ASCs [13]. Some of these cells secrete chemokines (e.g., CCL2/MCP-1), cytokines (e.g., IL-4, IL-13) and growth factors (e.g., VEGF, CSF1/M-CSF and CSF2/GM-CSF) that trigger the recruitment of monocytes, and can promote cancer progression through the activation of tumor-associated macrophages (TAMs) [14, 15]. TAMs comprise 50–80% of the tumor mass, and their presence often correlates with a poor prognosis in most cancers [16–18]. It was long thought that TAMs present with an anti-inflammatory phenotype that is associated with their pro-tumoral activity. Now, however, it is generally accepted that they have a mixed polarization phenotype, sharing both anti-inflammatory/pro-fibrotic and pro-inflammatory properties [19–21].

Several factors of the tumor microenvironment are known to influence macrophage properties. For example, it has recently been shown that ASCs may skew the phenotype of macrophages from a pro-inflammatory to an anti-inflammatory state via soluble factors [18, 22–24]. While the molecular mechanisms underlying this crosstalk, particularly in the context of cancer, remain largely obscure, it has been described that ASCs isolated from subjects with obesity can foster tumor cell progression by inducing epithelial-to-mesenchymal transition (EMT), enhancing invasion and migration capacities, and by triggering proliferation and metastasis [2, 10, 25, 26]. In this line, we recently demonstrated that the hostile environment of chronic inflammation associated with obesity may alter the functional and immunological properties of ASCs [27, 28]. More specifically, we showed that obese-derived ASCs (obASCs) secrete high levels of survivin [29], a member of the inhibitor of apoptosis family of proteins that is generally considered to be a tumor progression marker [30], and is involved in cell apoptosis, proliferation, division and senescence [31–34]. Survivin was initially believed to be absent in normal adult tissues and to be overexpressed in fetal and tumor cells [35, 36], but accumulating evidence suggests that it also plays critical roles in the survival, proliferation and differentiation of normal cells, including activated lymphocytes, macrophages and ASCs [29, 37–40].

Here, we show that the obASC secretome – the collection of proteins secreted into the extracellular space – modulates macrophage plasticity and generates a mixed-polarization phenotype with tumor-promoting properties. Importantly, our findings suggest that survivin serves as a new molecular link in the interplay between ASCs and macrophages in the tumor microenvironment and shed light on a novel mechanism by which obesity may sustain cancer development and progression.

## Materials and methods

### Cell lines and culture conditions

The human monocyte leukemia cell line THP-1, and the embryonic kidney HEK-293, liver cancer HepG2 and colonic cancer Caco2 and HT29 cell lines were obtained from the ATCC (Rockville, MD, USA). To obtain macrophages, THP-1 cells were cultured in Roswell Park Memorial Institute (RPMI)-1640 medium supplemented with 10% fetal bovine serum (FBS), 1% antibiotics/antimycotics (penicillin, streptomycin and fungizone solution) and 0.1 μg/ml phorbol myristate acetate (PMA; Sigma-Aldrich, St Louis, MO, USA) for 72 h at a density of 85,000 cells/cm^2^. Adherent (non-stimulated) cells (M0) were subsequently cultured in the same medium without PMA for 24 h. HEK-293, Caco2 and HT29 cells were cultured in Dulbecco’s Modified Eagle’s Medium (DMEM) supplemented with 10% FBS and 1% antibiotics/antimycotics at a density of 12,000, 6,600 and 20,000 cells/cm^2^, respectively. HepG2 cells were propagated in DMEM/F12 supplemented with 1% L-glutamine (Sigma-Aldrich), 10% FBS, 1% antibiotics/antimycotics solution and 2% HEPES (HyClone, Logan, UT, USA) at a density of 40,000 cells/cm^2^. All cells were culture in a humidified incubator at 37 °C with 5% CO_2_. Experiments using conditioned medium (CM) were performed using 24 h cultures.

### Isolation and culture of ASCs

ASCs were isolated from VAT of subjects with or without obesity undergoing nonacute surgical interventions, such as hernia or cholecystectomy, in a scheduled routine surgery following published protocols [27–29]. Donors included 12 lean (body mass index [BMI] = 22.2 ± 2.3, 66% female, age = 47.2 ± 7.3 years) and 11 obesity (BMI = 31.4 ± 1.8, 55% female, age = 45.7 ± 6.7 years) individuals. Briefly, VAT was washed extensively with phosphate buffered saline (PBS) to remove debris and next treated with 0.2% collagenase type I (Sigma-Aldrich) in PBS and 1% bovine serum albumin for 1 h at 37 °C with gentle agitation. Digested samples were centrifuged at 300×g at 4 °C for 5 min to separate adipocytes from stromal cells. The pellet containing the stromal cell fraction was resuspended in stromal culture medium consisting of DMEM/F12 with 10% FBS and 1% antibiotics/antimycotics solution and cultured at 37 °C in a humidified incubator with 5% CO_2_. To prevent spontaneous differentiation, primary cultures of ASCs at passage 0 (P0) were grown to 90% confluence and harvested with trypsin-EDTA. At P3, the minimal functional and quantitative criteria established by the International Society of Cell Therapy (ISCT) and the International Federation for Adipose Therapeutics and Science (IFATS) were determined by flow cytometry, as described before [27]. CM was collected at P3–7 after 24 h in culture using a minimum concentration of ASCs of 10,000 cells/cm^2^ and centrifuged at 400×g for 5 min. See supplementary Fig. 1 for the different experimental designs.

### Macrophage differentiation

To polarize THP-1 macrophages into pro- or anti-inflammatory macrophages, *Escherichia coli*-derived lipopolysaccharide (LPS) (250 ng/ml; serotype 0111:B4, number L4391, Sigma-Aldrich) or human recombinant IL-4 (50 ng/ml; PeproTech, Rocky Hill, NJ, USA) was added, respectively, to the cultures overnight.

### Adenovirus infection

Adenoviruses expressing WT or T34A-mutant survivin were added to HEK-293 cells or M0-macrophages at a multiplicity of infection (MOI) of 50, followed by incubation for 2 h at 37 °C in Opti-MEMTM Medium (Gibco; Thermo Fisher Scientific Inc., Waltham, MA, USA). An adenovirus expressing GFP was used as control. After incubation, adenovirus-containing medium was replaced with standard culture medium. Cells and CM for antibody neutralizing experiments were collected after 24 h of incubation.

### Co-culture assays

Adenovirus-infected HEK-293 cells were cultured in DMEM in the upper compartment of a two-chamber Transwell system (#3412; Corning, Costar, Cambridge, MA, USA). THP-1 macrophages were differentiated in the bottom well of the chamber in complete RPMI-1640 medium (adherent). Cells were co-cultured for 24 h at 37 °C with 5% CO_2_. In parallel, each cell type was grown individually at the same cell concentration as for co-culture, in similar plates.

For triple co-culture experiments, THP-1 cells were cultured in RPMI-1640 with PMA in the upper Transwell compartment. After adherence, THP-1 macrophages were activated with CM from lean (ln) ASCS or obASCs or co-cultured with lnASCs or obASCs overnight. Next, the medium was replaced by RPMI-1640 and CM-activated macrophages were co-cultured with HepG2 cells in the bottom well of the chamber for 24 h at 37 °C with 5% CO_2_. See supplementary Fig. 1 for the different experimental designs.

### Transwell migration and invasion assays

The migratory capacity of cancer cells in response to 24 h application of CM from macrophages overexpressing survivin or co-culture with THP-1 macrophages and ASCs was determined using 24-well Transwells (#3422, Costar) as previously described [28]. In total, 2 × 10^5^ cancer cells were suspended in 200 μl CM and added to the upper chamber, and 500 μl culture medium was placed in the lower chamber. Invasion capacity was determined as for migration, except that the membrane was first coated with Matrigel® (0.7–0.9 mg/ml; Sigma-Aldrich) in PBS for 2 h at 37 °C. After 24 h incubation, the cells in the upper compartment were removed using cotton swabs after which the cells on the lower surface of the membrane were fixed in 4% glutaraldehyde, stained with 2% toluidine blue and counted.

### Cell fractionation assay

THP-1 macrophages were lysed in homogenization buffer (20 mM Tris-HCl, 2 mM EDTA, 2 mM EGTA, 1 mM PMSF, 10 mM 2-mercaptoetanol, 10 μg/ml aprotinin and 10 μg/ml leupeptin) followed by mechanical disruption using a Dounce homogenizer. Total lysates were centrifuged at 1300 rpm for 10 min after which the pellets containing nuclei were collected. Supernatants containing cytoplasmic fractions were centrifuged at 13,000 rpm for 1 h.

### Survivin blocking experiments

Survivin neutralization was performed by adding 20 μg/ml of an anti-survivin antibody (ab76424; Abcam, Milton, Cambridge, UK) for 1 h at room temperature before adding the medium to THP-1 or HepG2 cells. A negative epitope control (Rabbit IgG Isotype Control, Invitrogen) was included in each experiment.

### Gene expression analysis

Total RNA was extracted from cells using TRI Reagent (Molecular Research Center, Cincinati, OH, USA). Quantification was performed at 260 nm and purity was assessed by the OD260/OD280 ratio. For gene expression analysis, 1 μg RNA was reverse-transcribed with random primers using a Reverse Transcription System (Applied Biosystems, Foster City, CA, USA). Quantitative RT-PCR (qRT-PCR) was conducted on a ProFlex PCR System using TaqMan Gene Expression Assays (Applied Biosystems) (Supplementary Table 1). Results were calculated using the comparative Ct method (2-ΔΔCt) normalized to the expression of the housekeeping gene 18S (Hs 03928985_g1) and expressed relative to the control condition set to 1. Two technical duplicates were performed for each biological replicate.

### Protein expression analysis

Cells were lysed in Mammalian Protein Extraction Reagent (M-PER™, Thermo Fisher Scientific) containing a protease and phosphatase inhibitor cocktail (Sigma-Aldrich), and equal amounts of protein were separated on SDS-PAGE gels, transferred to Immobilon membranes (Merck Millipore, Burlington MA, USA) and blocked. Immunoreactive bands were visualized using a SuperSignal West Femto chemiluminescent substrate (Pierce, Rockford, IL, USA) and images were captured on a VersaDoc imaging system equipped with Quantity One software (Bio-Rad, Hercules, CA, USA). The following antibodies (diluted 1:1000) were used: anti-survivin (ab76424; Abcam, Milton, Cambridge, UK); anti-survivin, anti-IL-1β, anti-CD14, anti-TGF-β, anti-Stat6 and anti-phospho-Stat6 (Tyr641) (#2808, #12703, #56082, #3711, #9362 and #56554, respectively; Cell Signaling Technologies, Danvers MA, USA); anti-HIF-1α and anti-PPARγ (sc-10,790 and sc-7196, respectively; Santa Cruz Biotechnology PaloAlto, CA, USA); anti-phospho-survivin (Ser20) (NB110–92717; Novus Biologicals, Littleton, CO, USA); anti-phospho-survivin (Thr34) and anti-β-tubulin (PA1–16853 and MA5–16308, respectively; Thermo Fisher Scientific); and anti-lamin A and anti-GAPDH (L1293 and MA5–15738, respectively; Sigma-Aldrich). Secondary peroxidase-conjugated antibodies used (diluted 1:2000) were as follows: anti-rabbit and anti-mouse (NA934 and NXA931, respectively; GE Healthcare, Chicago, IL, USA) and anti-chicken (ab131366; Abcam).

### Immunofluorescence assay

THP-1 macrophages grown on coverslips were fixed with 4% (*w*/*v*) paraformaldehyde, rehydrated with 2% (*v*/v) fish skin gelatin and permeabilized with 0.2% Triton X-100 prior to incubation with 5% (v/v) goat serum. Subsequently, cells were incubated overnight at 4 °C with an anti-survivin antibody (1:500 dilution; ab76424, Abcam) in PBS containing 1% goat serum. Next, the cells were washed with PBS and incubated for 1 h at room temperature with an Alexa Fluor 488-conjugated secondary antibody (1:100 dilution; Life Technologies, Carlsbad, CA, USA) followed by mounting with ProLong Diamond Antifade Mountant with DAPI (Invitrogen). Images were acquired on a Leica DM 4000B fluorescence microscope (Leica Microsystems, Wetzlar, Germany) and captured with a Leica DFC 300 FX camera (Leica Microsystems). Fluorescence intensity was analyzed using ImageJ software (https://imagej.nih.gov/ij/download.html), as described before [41].

### Immunohistochemistry

Paraffin-embedded cellular blocks from 16 ascitic fluid samples were selected from the Pathology Department of the Hospital Verge de la Cinta, Tortosa, Tarragona, Spain. Hematoxylin and eosin (HE)-stained slides for all cases were reviewed by a pathologist to confirm the diagnosis. To obtain homogeneity and reproducibility in staining, a tissue microarray (TMA) was generated with two representative cylinders from the original paraffin block of each case. Cylinders were carefully selected by the pathologist and transferred into ready-made holes in a paraffin block using the Arraymold tool. For immunohistochemistry, the TMA block was sectioned at a thickness of 4 μm and each slide was deparaffinized in xylene for 20 min, rehydrated in a decreasing ethanol series and washed with PBS. The sections were heated at 96 °C for 20 min for antigen retrieval and then incubated with appropriate primary antibodies directed against survivin (Clone 12C4), Ber-Ep4 (Clone Ber-EP4), CD68 (Clone KP1) or calretinin (Clone DAK-Calret 1) (all from DAKO Corp., Carpenteria, CA, USA). Automatic immunodetection was performed by the ENDVISIONTM FLEX method (DAKO) using 3,3′-diaminobenzidine chromogen as substrate and counter-staining with hematoxylin. The presence or absence of tumor cells was confirmed by positive Ber-Ep4 staining, the presence of macrophages was confirmed by positive CD68 staining, and the presence of mesothelial cells was confirmed by positive calretinin staining.

Immunostained cylinders were evaluated by two blinded observers and the subcellular localization of survivin detected in the tumor cells and macrophages was scored semi-quantitatively for staining intensity and percentage of positive cells. The staining intensity was defined has weak (1+), moderate (2+) or strong (3+).

### Survivin concentration determination

Survivin concentrations were measured in the CM by ELISA (R&D Systems, Minneapolis, MN, USA).

### Statistical analysis

In vitro experiments were performed 3–4 times and pooled for statistical analysis. Data are presented as mean ± S.E.M., and represents the number of biologically-independent samples. Differences between groups were determined using unpaired Student’s t test to compare two groups (two-tailed, 95% confidence interval) and one-way analysis of variance (ANOVA) with Tukey’s multiple comparisons tests to compare three groups. The analyses were performed using GraphPad Prism 8.0.2 software (GraphPad Software Inc., La Jolla, CA, USA). A *p* value < 0.05 was considered statistically significant.

## Results

### Conditioned medium from obese-derived ASCs induces macrophage switching

**Fig. 1:**
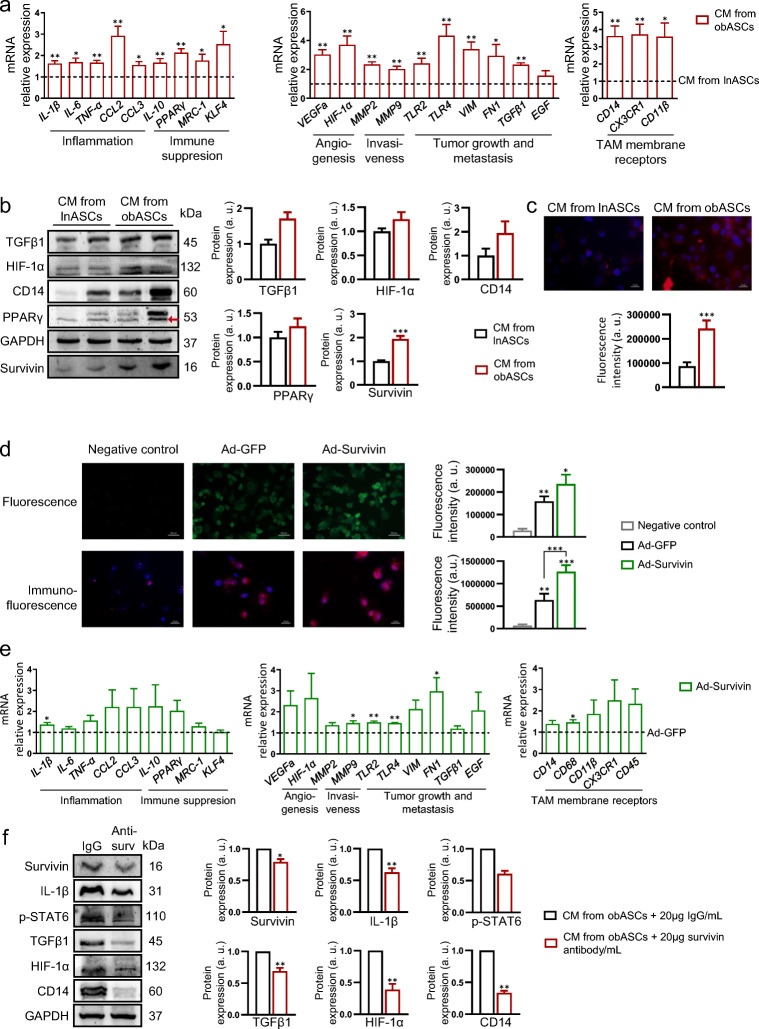
An obese environment changes the macrophage phenotype in a survivin-dependent manner. **a** Gene expression analysis of THP-1-derived macrophages cultured with conditioned medium (CM) from visceral ASCs of lean donors (lnASCs) or subjects with obesity (obASCs). mRNA CM lnASC values are arbitrarily set to 1.0 (dotted line). *n* = 5 in each group, *p < 0.05; **p < 0.01 versus CM from lnASCs. **b** Western blotting of TGFβ1, HIF-1α, CD14, PPARɣ and survivin in THP-1-derived macrophages cultured with CM from lnASCs or obASCs. GAPDH was used as a loading control. Shown is a representative image and densitometry analysis (arbitrary units), *n* = 8 for survivin and *n* = 2–4 for other proteins. ***p < 0.001 versus CM from lnASCs. **c** Immunofluorescence of survivin in THP-1-derived macrophages cultured with CM from lnASCs or obASCs. Survivin is marked in red and the cell nucleus is marked in blue (DAPI). Shown is a representative image and fluorescence quantification. ***p < 0.001 versus CM from lnASCs. **d** Fluorescence and immunofluorescence analysis of internalized survivin in THP-1-derived macrophages co-cultured with HEK-293 cells. In the fluorescence images green denotes the GFP-tag of the adenovirus. In the immunofluorescence images, survivin is marked in red and the cell nucleus is marked in blue (DAPI). Shown is a representative image and fluorescence quantification. **p < 0.01 versus negative control; ***p < 0.001 versus negative control and ad-GFP. **e** Gene expression analysis in THP-1-derived macrophages co-cultured with HEK-293 cells overexpressing control adenovirus (ad-GFP) or survivin (ad-survivin). mRNA ad-GFP values are arbitrarily set to 1.0. *n* = 3, *p < 0.05; **p < 0.01 versus co-culture with ad-GFP. **f** Immunoblotting analysis of THP-1-derived macrophages cultured with CM-obASC with or without an anti-survivin neutralizing antibody. Shown is a representative image and densitometry analysis (arbitrary units). *n* = 3–4, *p < 0.05; **p < 0.01 versus CM-obASC without anti-survivin antibody (IgG). Data information: All values are expressed as mean ± S.E.M. Statistical analyses: unpaired Student’s t test or ANOVA with Tukey’s multiple comparisons test

To test whether an obese microenvironment, specifically the obASC secretome, can modulate macrophage phenotypes, we cultured differentiated human THP-1 macrophages for 24 h with conditioned medium (CM) from visceral-derived ASCs isolated from subjects without obesity (lnASCs) or with obesity (obASCs) (Supplementary Fig. 1A), after which we surveyed the expression of a panel of genes related to immune regulation, TAM membrane receptors and cell invasion (Supplementary Table 1). We found that the expression of genes related to inflammation (IL-1β, IL-6, TNFα, CCL2 and CCL3), immune suppression (IL-10, PPARɣ and KLF4), angiogenesis (HIF-1α and VEGFα), invasiveness (MMP2 and MMP9), tumor growth and metastasis (TLR2, TLR4, VIM, FN1, TGFβ1 and EGF), and TAM membrane receptors (CD14, CD11β, CX3CR1 and CD45) was significantly higher in macrophages cultured with CM-obASC than in those cultured with CM-lnASC (Fig. 1a). Protein expression of several selected markers (TGFβ1, HIF-1α, CD14 and PPARɣ) was in accordance with mRNA expression (Fig. 1b).

Given our previous results showing high levels of secreted survivin by obASCs, we next focused on its potential modulating effects on macrophages. We first evaluated whether CM from lnASCs and obASCs differentially affect survivin expression in macrophages. We found that the survivin mRNA expression level was not significantly altered (1 ± 0.22 vs 1.90 ± 0.34, *p* = 0.056), but that the protein expression level was higher in macrophages cultured with CM-obASC than in those cultured with CM-lnASC (Fig. 1b-c), suggesting that extracellular survivin of the obASC secretome is internalized by macrophages. Indeed, it has previously been described that survivin is packaged into exosomes and can be released into the extracellular compartment [42, 43]. Thus, to test whether macrophages are able to take up survivin, we infected HEK-293 cells with adenoviruses expressing green fluorescent protein (GFP) or GFP-tagged survivin, and co-cultured these cells with THP-1-derived macrophages for 24 h using a Transwell system that physically separates the two cell types (Supplementary Fig. 1B). Subsequent immunofluorescence analysis showed that the survivin levels were elevated in THP-1-differentiated macrophages co-cultured with HEK-293 cells expressing GFP-tagged survivin relative to the GFP control (Fig. 1d). Consistent with the results of the CM-obASC analysis (Fig. 1a), internalization of survivin by THP-1 macrophages was accompanied by an increase in the expression of immune regulatory genes, both pro- and anti-inflammatory (IL-1β), genes related to angiogenesis, invasiveness, tumor growth and metastasis (MMP9, TLR2, TLR4 and FN1) and to TAM membrane receptors (CD68) (Fig. 1e). To test whether the effect of obASCs on the macrophage phenotype is dependent on secreted survivin, we neutralized survivin using a specific anti-survivin antibody (Supplementary Fig. 1C). We found that the expression of survivin, IL-1β, p-STAT6, TGFβ1, HIF-1α and CD14 in macrophages cultured with CM-obASC was significantly lower when the conditioned medium was first treated with the antibody (Fig. 1f).

Overall, these data indicate that an obese microenvironment generates “hybrid” macrophages that exhibit characteristics of both pro- and anti-inflammatory activation states, suggestive of a pro-tumoral phenotype, likely mediated by a survivin-dependent mechanism.

### Expression and intracellular localization of survivin in macrophages

**Fig. 2:**
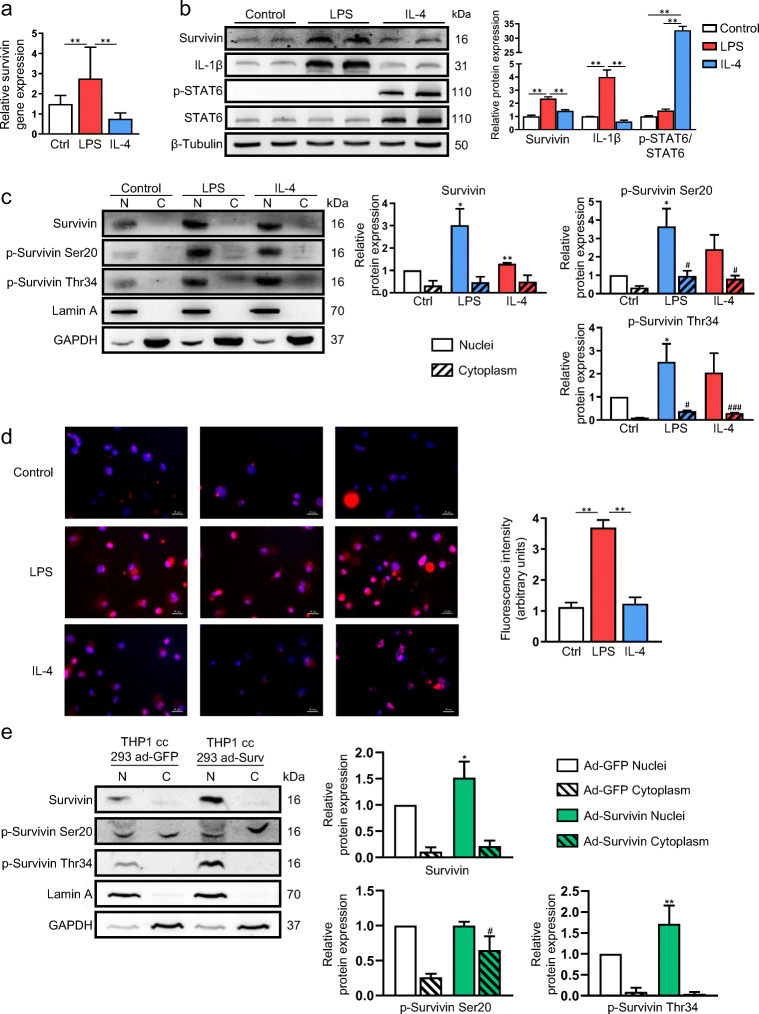
Survivin expression is elevated in activated macrophages. **a** Survivin gene expression in THP-1-derived macrophages treated with LPS or IL-4. *n* = 4 in each group. **b** Survivin protein expression in THP-1-derived macrophages polarized with LPS or IL-4. IL-1β and p-STAT6 were used to confirm polarization and β-tubulin was used as a loading control. Representative image and densitometry analysis (arbitrary units). *n* = 4 in each group. **c** Survivin localization by immunoblotting of THP-1-derived macrophages polarized with LPS or IL-4. Lamin A and GAPDH were used as nuclear (N) and cytoplasmic (C) markers, respectively. Representative image and densitometry analysis (arbitrary units). *n* = 3. **d** Immunofluorescence staining of survivin (red) and DAPI (blue) in control THP-1-derived macrophages and in LPS- or IL-4-polarized macrophages. Representative image and densitometry analysis (arbitrary units). *n* = 6. **e** Western blotting of THP-1-derived macrophages co-cultured with HEK-293 cells overexpressing GFP or survivin. Representative image and densitometry analysis (arbitrary units). *n* = 3. Data information: All values are expressed as mean ± S.E.M. In (**a–d**), *p < 0.05; **p < 0.01; **p < 0.01 versus inactivated macrophages (control), versus LPS-activated macrophages or versus nuclei of inactivated macrophages; ^#^*p* < 0.05; ^###^*p* < 0.001 versus cytoplasm of inactivated macrophages. In **e,** *p < 0.05; **p < 0.01 versus ad-GFP nuclei; ^#^*p* < 0.05 versus ad-GFP cytoplasm. Statistical analyses: unpaired Student’s t test

To further explore the potential role of survivin in macrophage plasticity, we first tested whether survivin expression was regulated in THP-1 macrophages under pro- and anti-inflammatory stimuli, i.e., LPS and IL-4, respectively. We found that survivin mRNA (Fig. 2a) and protein (Fig. 2b) expression were significantly higher in LPS (classically)-activated macrophages than in IL-4 (alternatively)-activated macrophages. Successful polarization of activated macrophages was confirmed by measuring the expression of IL-1β (a pro-inflammatory protein) and phosphorylated-STAT6 (an anti-inflammatory signaling marker) (Fig. 2b). This result is consistent with published transcriptome data (GEO GSE27792 http://www.ncbi.nlm.nih.gov/geo/), showing that survivin expression is higher in human pro-inflammatory macrophages polarized by granulocyte-macrophage colony-stimulating factor (GM-CSF) than in anti-inflammatory macrophages polarized by macrophage colony-stimulating factor (M-CSF).

Because the multiple functions of survivin have been closely tied to its subcellular localization and post-translational modification [44, 45], we next analyzed protein expression of total and phosphorylated survivin (at Ser20 and Thr34) in both nuclear and cytoplasmic fractions. Using subcellular fractionation experiments, we found that total survivin was predominantly expressed in the nucleus of macrophages and increased in macrophages activated with LPS and IL-4 relative to M0-macrophages (control) (Fig. 2c). We also detected increased survivin phosphorylation on Ser20 and on Thr34 in the nucleus of activated macrophages (LPS and IL-4) relative to controls, and increased expression of phosphorylated survivin at Thr34 in the cytoplasm of activated macrophages versus controls (Fig. 2c). Immunofluorescence analysis further confirmed the increased survivin expression in activated macrophages (Fig. 2d).

Subcellular fractionation experiments also showed that when macrophages sequestered survivin from the extracellular medium – in this case THP-1 macrophages co-cultured with HEK-293 cells overexpressing survivin – the nuclear localization of survivin was maintained (Fig. 2e and Supplementary 1B).

Taken together, these results point to nuclear survivin as a potential key factor for macrophage phenotype and functionality.

### Survivin alters the macrophage phenotype and enhances pro-tumoral activity

**Fig. 3:**
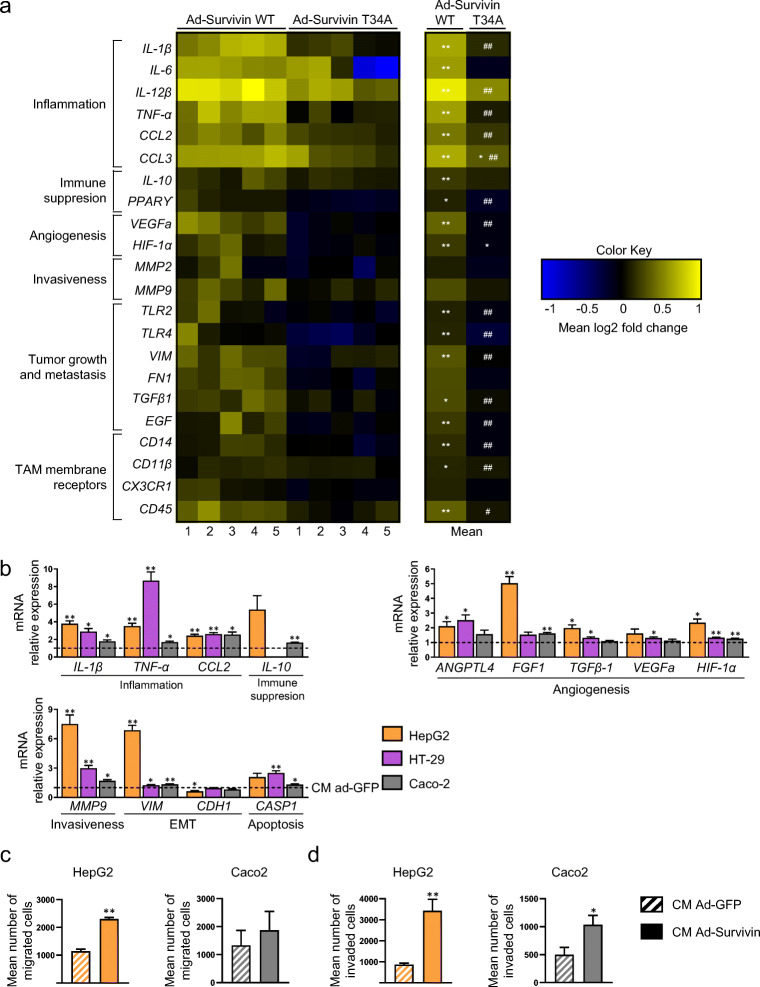
Survivin overexpression in macrophages promotes a tumor-associated macrophage phenotype and alters gene expression patterns and migratory and invasive capacities of cancer cells. **a** Heatmap of gene expression in THP-1-derived macrophages infected with a recombinant adenovirus expressing GFP (ad-GFP), wild-type (WT) survivin or mutated (T34A) survivin. mRNA ad-GFP values are arbitrarily set to 1.0. *n* = 5, one-way ANOVA with Tukey’s multiple comparisons, *p < 0.05; **p < 0.01 versus ad-GFP; ^#^*p* < 0.05; ^##^*p* < 0.01 versus ad-survivin WT. **b** Gene expression in HepG2, HT-29 and Caco2 cells cultured with conditioned medium (CM) from ad-GFP- or ad-survivin-infected THP-1-derived macrophages. mRNA ad-GFP values are arbitrarily set to 1.0. *n* = 4 in each group. **c**, **d** Transwell cell migration (c) and invasion (d) assays of cancer cells cultured with CM from ad-GFP- and ad-survivin-infected THP-1-derived macrophages. Striped columns are cells cultured with CM from ad-GFP-infected macrophages, plain columns are cells cultured with CM from ad-survivin-infected macrophages. *n* = 3 in each group. Data information: In (b–d), data are presented as mean ± SEM. *p < 0.05; **p < 0.01 versus CM from ad-GFP-infected macrophages (paired Student’s t test)

To test whether survivin plays a role in macrophage phenotype and functionality, we compared adenoviral overexpression of wild-type (WT) survivin with a non-phosphorylatable mutant (T34A) (Supplementary Fig. 1D), which has been reported to act as a dominant-negative protein by competing with endogenous survivin for access to kinases, thereby preventing the phosphorylation of WT protein [29, 46]. Transduction of WT survivin into THP-1 macrophages (M0) led to its expression in both the nucleus and the cytoplasm (Supplementary Fig. 2), concomitant with a significant increase in the expression of genes related to inflammation (e.g., IL-1β, IL-6, TNFα, CCL2 and CCL3), immune suppression (e.g., IL-10 and PPARɣ), angiogenesis (e.g., HIF-1α and VEGFα), invasiveness (e.g., MMP9), tumor growth and metastasis (e.g., TLR2, TLR4, VIM, FN1, TGFβ1 and EGF) and TAM membrane receptors (e.g., CD14, CD11β and CD45) (Fig. 3a), again reflecting a mixed pro/anti-inflammatory phenotype with potential pro-tumoral activity. These results are consistent with those of the impact of CM-obASC on the same cells (Fig. 1a). By contrast, we found that overexpression of T34A survivin had no effect on the macrophage phenotype (Fig. 3a), suggesting that the effect of survivin on macrophage phenotype regulation is dependent on its phosphorylation at Thr34.

To further assess whether survivin-overexpressing macrophages may have pro-tumoral activity, we repeated the CM experiments on cancer cells (Supplementary Fig. 1E). We found that CM from survivin-overexpressing THP-1 macrophages significantly modulated gene expression patterns in both human liver carcinoma cells (HepG2) and human colorectal cells (HT29 and Caco2), specifically of those involved in inflammation, angiogenesis, EMT and invasiveness (Fig. 3b). In line with these observations, we found that the migratory and invasive capacities of HepG2 and Caco2 cells were increased when they were cultured in CM from survivin-overexpressing macrophages compared to those from GFP-overexpressing control cells (Fig. 3c and d).

Overall, these data indicate that survivin may skew the phenotype and functionality of macrophages towards a TAM-type profile.

### Macrophages are a source of survivin in the tumor microenvironment

**Fig. 4:**
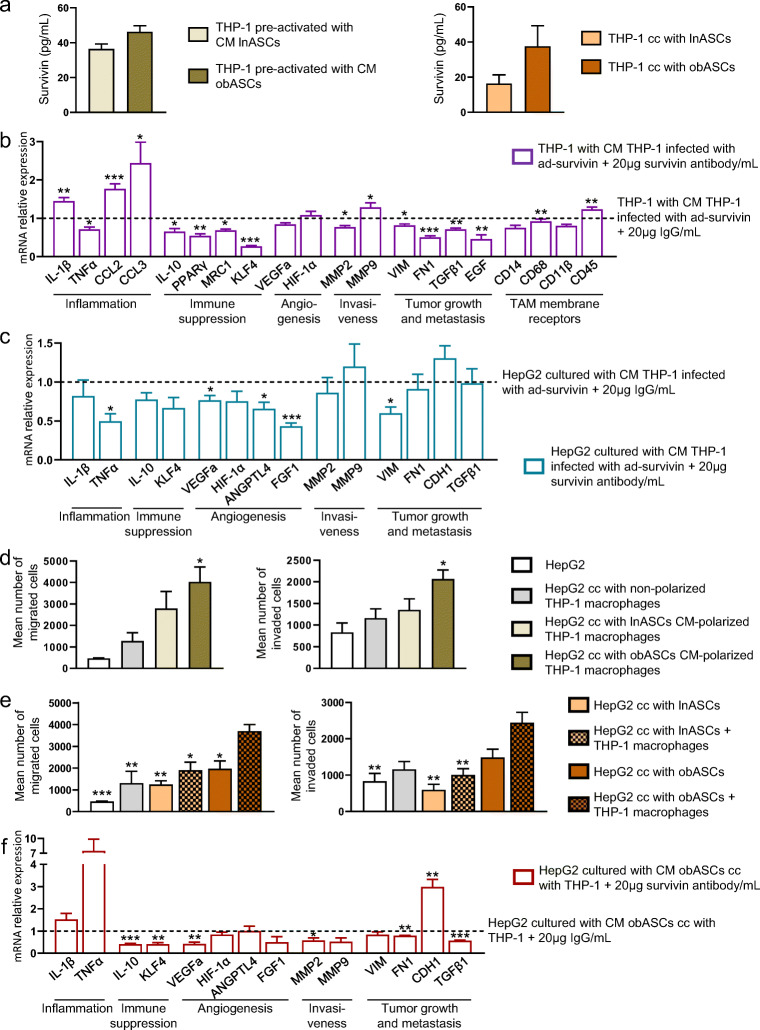
Macrophages are a source of survivin in the tumor microenvironment. **a** Survivin secretion in THP-1-derived macrophages cultured in conditioned medium (CM) of obese or lean ASCs (left) and co-cultured with lean or obese ASCs (right). *n* = 3. **b** Gene expression analysis in THP-1-derived macrophages cultured in CM of survivin-overexpressing macrophages treated with a specific survivin-neutralizing antibody or an IgG (control). Control values are arbitrarily set to 1.0 (dotted line). *n* = 6. **c** Gene expression analysis of HepG2 cells cultured in CM of survivin-overexpressing macrophages treated with a specific survivin-neutralizing antibody or an IgG (control). Control values are arbitrarily set to 1.0 (dotted line). *n* = 4. **d** Transwell cell migration and invasion assays of HepG2 cells cultured in (i) control media, or co-cultured with THP1-macrophages (ii) non-activated, pre-activated with (iii) CM of lnASCs and (iv) CM of obASCs. *n* = 2 for control HepG2 and co-cultured with non-activated THP1-macrophages, *n* = 5 for HepG2 co-cultured with pre-activated THP1-macrophages with CM from ASCs. **e** Transwell cell migration and invasion assays of HepG2 cells (i) cultured in control media, co-cultured with (ii) THP1-macrophages, (iii) lean ASCs (lnASCs), (iv) lnASCs and THP1-macrophages, (v) obese ASCs (obASCs) and (vi) obASCs and THP1-macrophages. *n *= 2 for control HepG2 and co-cultured with non-activated THP1-macrophages, *n* = 5 for HepG2 co-cultured with ASCs and THP1-macrophages with ASCs. **f** Gene expression analysis in HepG2 cells cultured in CM of THP-1 macrophages pre-activated with CM-obASCs and treated with a survivin-neutralizing antibody or IgG (control). Control values are arbitrarily set to 1.0 (dotted line). *n *= 4. Data information: All values are expressed as mean ± S.E.M. Statistical analyses: unpaired Student’s t test or ANOVA. In (b, c, f) *p < 0.05; **p < 0.01; ***p < 0.001 versus CM + 20 μg /ml IgG. In (d) *p < 0.05 versus HepG2. In (e) *p < 0.05; **p < 0.01; ***p < 0.001 versus HepG2 with obASCs + THP-1 macrophages

To gain further insight into the role of the different cell types in the tumor microenvironment, we first investigated macrophage-secreted survivin. To this end, THP-1 macrophages were cultured with either CM-obASC or CM-lnASC, or co-cultured with either lean or obese ASCs. We observed that “obesity” potentiated the release of survivin by macrophages (Fig. 4a). To test whether the survivin released by macrophages can modulate their phenotype, we incubated THP-1 macrophages with CM from survivin-overexpressing macrophages treated with a survivin-neutralizing antibody (Supplementary Fig. 1F). We found that neutralizing extracellular survivin aggravated macrophage inflammation (as measured by the expression of its associated genes), but significantly decreased the expression of genes related to immune suppression (e.g., IL-10, PPARɣ, MRC1 and KLF4), invasiveness (e.g., MMP2), tumor growth and metastasis (e.g. VIM, FN1, TGFβ1 and EGF) and TAM-specific markers (e.g., CD68) (Fig. 4b), suggesting that an autocrine survivin loop reprograms the TAM phenotype. To test the paracrine effect of macrophage-secreted survivin, HepG2 cells were cultured with CM of survivin-overexpressing macrophages treated with a survivin-neutralizing antibody (Supplementary Fig. 1F). Blocking survivin reversed the tumoral phenotype induced by macrophage-derived survivin (Fig. 3b) and significantly down-regulated the expression of inflammation-, angiogenesis- and EMT-related genes (Fig. 4c).

To better understand the role of survivin in the complex relationship between ASCs, macrophages and tumor cells, we performed triple co-culture experiments (Supplementary Fig. 1G-H). HepG2 cells co-cultured with macrophages and ASCs showed greater migration and invasion abilities than cells cultured with ASCs or macrophages alone, and this was more pronounced when obASCs were used (Fig. 4d and e). These effects were observed both in cancer cells co-cultured with obASC CM-polarized THP-1 cells (Fig. 4d, Supplementary Fig. 1G) and in cancer cells in triple co-culture (Fig. 4e, Supplementary Fig. 1H).

To confirm that these effects are survivin-dependent, we cultured cancer cells in survivin-neutralized CM collected from obASC/macrophage co-cultures and from pre-activated-macrophages cultured in CM-obASC (Fig. 4f, Supplementary Fig. 1I). Blocking survivin potentiated the inflammatory status of cancer cells (as indicated by an increase in IL-1β and TNFα expression), but significantly reduced the expression of immune suppression, angiogenesis, invasiveness and tumor growth-related markers, indicating that tumor microenvironment survivin (secreted by both obese ASCs and macrophages) promotes a pro-tumorigenic gene expression profile in HepG2 cells.

Overall, our data show synergistic actions of obASCs- and macrophage-derived survivin in enhancing the malignancy of cancer cells.

### Survivin is overexpressed in ascites-associated macrophages from patients with a malignant diagnosis

**Fig. 5:**
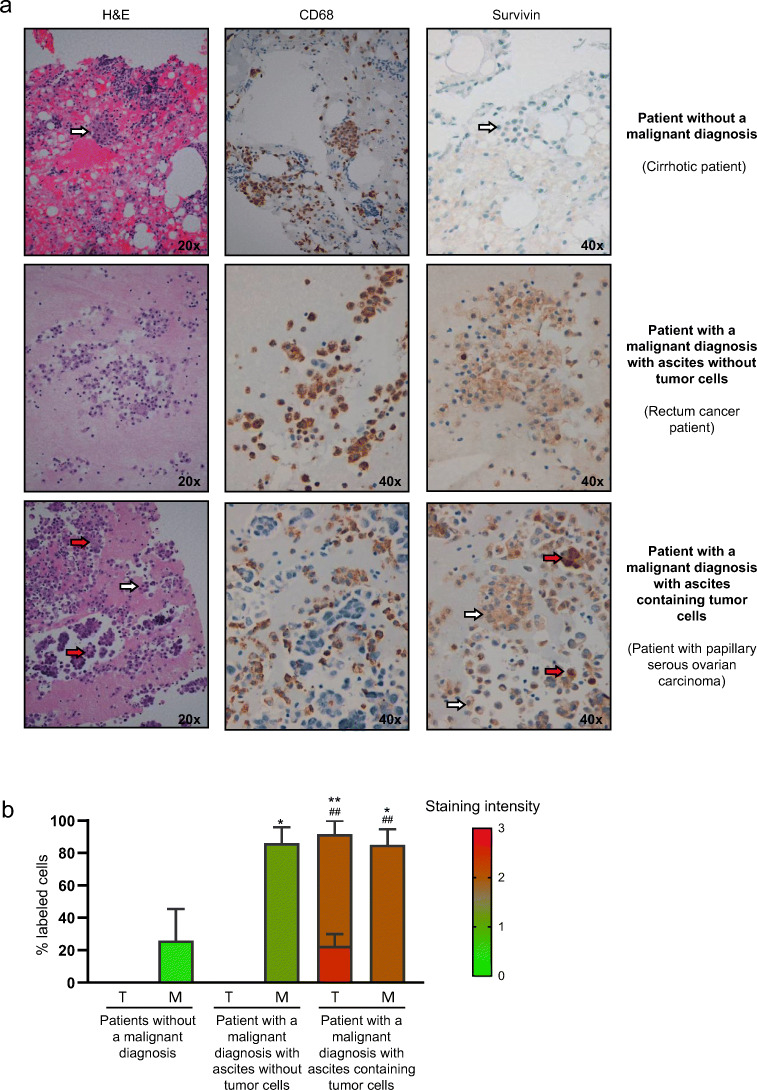
Survivin is overexpressed in ascites-associated macrophages from patients with a malignant diagnosis. **a** Representative images of ascitic fluid from patients without a malignant diagnosis (liver cirrhosis), patients with a malignant diagnosis with ascites without tumor cells (rectal cancer) and patients with a malignant diagnosis with ascites containing tumor cells (papillary serous ovarian carcinoma). Hematoxylin and eosin (H&E) staining was performed to verify the diagnosis. CD68 immunostaining confirmed macrophage accumulation in ascitics fluid. Survivin immunostaining is mainly present in patients with a malignant diagnosis. Red arrows point to cancer cells, white arrows point to macrophages. **B** Percentage of cells labeled with survivin in tumoral cells (T) and macrophages (M) in each group of patients. The colour of the bars reflects the staining intensity, with 0 null intensity and 3 the highest intensity. Bars represent the localization of survivin in the cytoplasm, except tumoral cells, which also show a percentage of survivin in the nucleus. Data information: *n* = 6 in malignant ascites and *n* = 5 in benign ascites. All values are expressed as mean ± S.E.M. Statistical analyses: ANOVA and Tukey’s multiple comparisons tests, *p < 0.05; **p < 0.01 for % labeled cells; ^##^*p* < 0.01 for staining intensity versus cytoplasmic staining (percentage and intensity) of patients without a malignant diagnosis

Although a variety of macrophage subsets has been reported to coexist in human tumors, their phenotypes are still not fully defined [47], highlighting the need to uncover unique biomarkers for this specific macrophage population. Based on our results on survivin as a possible regulator of the TAM phenotype, we hypothesized that it could also serve as a TAM biomarker. To test this, we analyzed survivin expression by immunochemistry in ascites from 11 patients without obesity but with different tumors (stomach, serous ovarian carcinoma, ovarian clear cell, rectum, bile duct, colon and pleural mesothelioma) and from 5 patients without obesity and without tumors (cirrhosis, congestive heart failure and ovarian cystadenoma with mesothelial hyperplasia). Ascites is defined as a pathological fluid accumulation within the abdominal cavity, which may be a complication associated with a number of different diseases, including cancer [48]. Cancer-associated ascites may contain tumor cells and immune cells, including macrophages. These peritoneal macrophages play an indispensable role in cancer progression [49] and, therefore, ascites may be a rich source of TAMs. We classified our patient cohort into three groups: 6 patients with a malignant diagnosis with ascites containing tumor cells, 5 patients with a malignant diagnosis with ascites without tumor cells and 5 patients without a malignant diagnosis (Table 1). As expected, we found a high abundance of macrophages (CD68+) in ascitic fluid in all groups (Fig. 5a, shown is a representative image for each group).

**Table 1:** Survivin expression in tumor cells and macrophages of ascites from patients with different tumors and patients without tumors

Diagnosis	Tumor cells		Macrophages	
	Staining intensity Nucleus / cytoplasm	% Labeled cells Nucleus / cytoplasm	Staining intensity Nucleus / cytoplasm	% Labeled cells Nucleus / cytoplasm
Patients with a malignant diagnosis with ascites containing tumor cells				
ADC/Stomach	3+ / 2+	10% / 100%	0 / 2+	0% / 100%
ADC/Serous ovarian carcinoma	2+ / 2+	20% / 100%	0 / 1+	0% / 50%
ADC/Serous ovarian carcinoma	3+ / 2+	40% / 100%	0 / 2+	0% / 100%
ADC/Ovarian clear cell	2+ / 1+	8% / 50%	0 / 1+	0% / 60%
ADC/Rectum	2+ / 2+	50% / 100%	0 / 3+	0% / 100%
ADC/Bile duct	3+ / 3+	8% / 100%	0 / 3+	0% / 100%
Patients with a malignant diagnosis with ascites without tumor cells				
ADC/Rectum	–	–	0 / 2+	0% / 100%
ADC/Serous ovarian carcinoma	–	–	0 / 1+	0% / 50%
ADC/Colon	–	–	0 / 1+	0% / 100%
ADC/Rectum	–	–	0 / 1+	0% / 100%
Pleural mesothelioma	–	–	0 / 1+	0% / 80%
Patients without a malignant diagnosis				
Cirrhosis	–	–	0 / 1+	0% / 100%
Cirrhosis	–	–	0 / 1+	0% / 30%
Cirrhosis	–	–	0 / 0	0% / 0%
Congestive heart failure	–	–	0 / 0	0% / 0%
Ovarian cystadenoma with mesothelial hyperplasia	–	–	0 / 0	0% / 0%

Data are presented as the percentage of positive cells by survivin (labeled cells) and scored semi-quantitatively for staining intensity. The staining intensity was defined as weak (1+), moderate (2+) or strong (3+)

Survivin expression was detected in virtually 100% of the tumor cells, which were only present in patients with a malignant diagnosis. Of the tumor cells positive for survivin, 92% showed survivin staining with a moderate-strong intensity in the cytoplasm, whereas 23% also showed survivin expression in the nucleus, with a strong intensity (Table 1, Fig. 5a and b). Remarkably, survivin was also detected in the cytoplasm of ascites-associated macrophages from patients with tumors (Fig. 5a). Specifically, the percentage of macrophages positive for survivin staining was similar between ascites with and without tumor cells (85% vs 86%), but the staining intensity was stronger in ascites containing tumor cells (Table 1 and Fig. 5). The absence of survivin in macrophages without a malignant diagnosis (Table 1 and Fig. 5) strongly indicates that survivin may serve as a specific biomarker of TAMs in ascites.

## Discussion

**Fig. 6:**
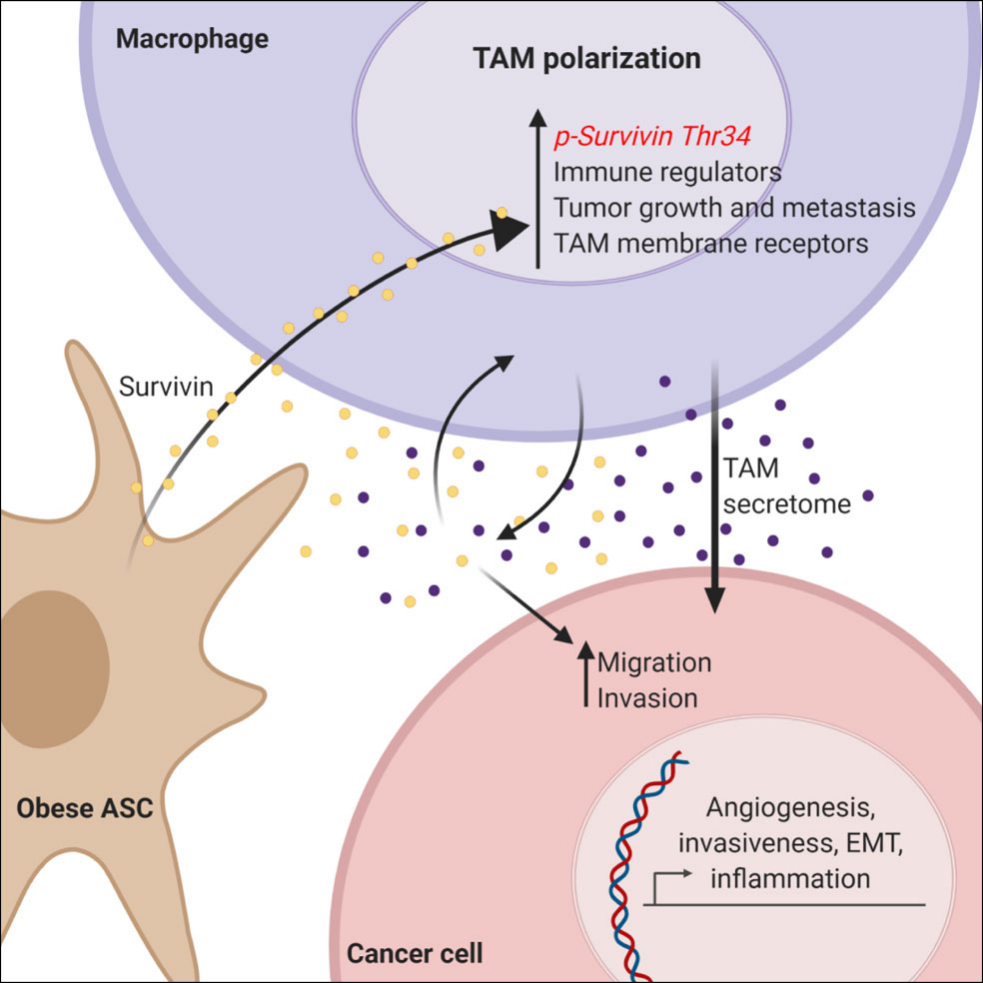
Graphical abstract. Obese adipose-derived stem cells modulate macrophage plasticity, promoting a mixed phenotype dependent on survivin. In the tumor microenvironment, obese adipose-derived stem cells induce pro-tumoral reprogramming of macrophages by a survivin-dependent mechanism. Macrophages internalize extracellular survivin, which drives macrophages towards a pro-tumor phenotype. Survivin-mediated polarization of macrophages is both dependent on its nuclear location and phosphorylation at Thr34. Pro-tumoral macrophages, also a source of survivin, further reinforce the malignant characteristics of cancer cells including epithelial-mesenchymal transition, migration and invasion

In the present study we provide evidence that survivin may play a role in reprogramming human macrophages towards a pro-tumoral phenotype. We found that, in the context of an obese environment, an enhanced secretion of survivin by ASCs provokes phenotypic and functional alterations in macrophages and, ultimately, enhances the malignancy of cancer cells (graphical abstract, Fig. 6).

A link between obesity and cancer has mostly been attributed to dysfunctional adipose tissue [50] and recent studies point to a potential role for ASCs in cancer progression [10, 51, 52]. Our findings implicate survivin as a new link between obesity and cancer, and lead us to propose a new survivin-dependent mechanism through which ASCs, specifically from patients with obesity, contribute to the pro-tumoral activation of TAMs. An obese environment increases tumor-recruitment of ASCs [10, 25, 51], which promote cancer cell growth, possibly via leptin secretion [10, 25]. However, indirect interactions, such as crosstalk between ASCs and other cells present in the tumor microenvironment, including TAMs, may also contribute to the complex network of processes at work within a tumor. Here, we uncovered survivin as a novel player in the ASC-TAM-cancer-cell crosstalk. It is well established now that TAMs orchestrate several critical processes related to tumor progression [53, 54], although several different TAM subsets have been described and there is currently no consensus regarding their phenotype [47, 55, 56]. Indeed, recent studies suggest that macrophages infiltrating the tumor microenvironment have a hybrid phenotype. TAMs can be considered as a unique in vivo macrophage population that shares some phenotypic characteristics with in vitro generated anti-inflammatory cells [19, 20] and metabolic similarities with in vitro produced pro-inflammatory macrophages [21]. Cleary, a better understanding of TAM function and phenotype will be necessary to fully comprehend their role in cancer progression.

Since macrophage diversity and plasticity are dependent on external stimuli, we focused on the impact of ASCs from patients with and without obesity in the programming of macrophages, in an attempt to further our understanding of the mechanisms by which obesity enhances tumorigenesis. We found that the secretome of ASCs alters the gene expression profile of macrophages by a mechanism that involves survivin. We previously demonstrated that obASCs can produce aberrant amounts of survivin [29]. Here we show that the effect of obASCs on establishing the macrophage phenotype is at least partially dependent on secreted survivin, but we cannot rule out the possibility that other factors from the ASC secretome also play a role. Survivin is an inhibitor of apoptosis highly expressed in most cancers and is associated with a more aggressive disease and a poor clinical outcome [57, 58]. In addition to its intracellular pro-cancer functions, extracellular survivin can be internalized and enhance cell aggressiveness by promoting proliferation while decreasing apoptosis [59]. Consistent with these findings, we here show that extracellular survivin within the tumor microenvironment increases the expression of inflammatory-, angiogenesis-, EMT- and invasiveness-related genes in cancer cells.

Beyond its role in proliferation and apoptosis, emerging data suggest that extracellular survivin may possess immunomodulatory properties in leukocytes [60]. Here, we show that macrophages are able to sequester extracellular survivin, which triggers a mixed phenotype with both pro- and anti-inflammatory properties. This internalization alters the expression of genes involved in angiogenesis, invasion, tumor growth and metastasis, and increases the expression of membrane receptors associated with the TAM phenotype. Consistent with these findings, we found that activated macrophages with both pro- and anti-inflammatory phenotypes exhibit elevated nuclear survivin levels and a higher phosphorylation on Thr34 (in the nucleus and cytoplasm). These data suggest that functional survivin may play an important role in macrophage activation. Indeed, we found that survivin-overexpressing macrophages have a gene expression profile similar to that of macrophages cultured with CM-obASCs. In addition, our results point to an autocrine survivin loop in macrophages to sustain their pro-tumoral phenotype. IL-4, which is abundant in the tumor microenvironment [61], increases survivin protein expression in human macrophages, which is in agreement with a recent report describing that survivin expression and location are regulated by IL-4 in colon cancer stem cells through a STAT6-dependent pathway [62]. Nonetheless, further studies are needed to comprehend the molecular mechanism by which survivin regulates macrophage polarization. Along this line, the multiple functions of survivin have been closely tied to its subcellular localization. It is widely acknowledged that nuclear survivin is involved in mitosis, whereas cytoplasmic surviving is related to apoptosis [63]. The finding that overexpression of mutated Thr34-survivin failed to impact macrophage gene expression indicates that the role of survivin in macrophage plasticity may be dependent on its phosphorylation on Thr34, which is usually linked to its anti-apoptotic function [46, 64, 65]. Nonetheless, no significant differences in apoptosis were detected when survivin was overexpressed or when macrophages were cultured with CM from obese ASCs. These data lead us to propose a new role for this post-translational modification in modulating the macrophage phenotype, specifically boosting a TAM profile.

The phenotype and function of TAMs in humans remain enigmatic. Access to human TAMs is limited, and most studies are restricted to using in vitro differentiated macrophages [66]. Moreover, the dearth of phenotypic markers for TAMs limits the potential of functional studies. The present study shows that obASCs may control a pro-tumoral program in macrophages in the tumor microenvironment through a survivin-dependent mechanism. Moreover, it identifies survivin as a specific biomarker of macrophages associated with malignant tumors, turning this protein into a potentially interesting clinical tool to identify malignancy, which may guide clinical decision-making.

In conclusion, the present study provides evidence for survivin as a stimulator of pro-tumorigenic polarization of TAMs. In addition, we uncover survivin as a key player in autocrine and paracrine loops between ASCs, macrophages and cancer cells that sustain tumorigenicity. We postulate that in an obesity setting, survivin secreted by ASCs may be internalized by macrophages, altering their function and phenotype and promoting tumor progression. Indeed, pro-tumoral macrophages may also contribute to higher levels of extracellular survivin in the tumor microenvironment, which may boost both migratory and invasive capacities of cancer cells (Fig. 6). While survivin is already a well-established target in experimental cancer therapy, our study offers a fresh perspective on its role in the interplay between cellular components of the tumor microenvironment. Further preclinical studies are warranted to confirm that targeting survivin in vivo can modulate the TAM phenotype and prevent cancer progression.

## Supplementary Information

ESM 1 (PDF 356 kb)

ESM 2 (PDF 73 kb)

ESM 3 (DOC 46 kb)

## Abbreviations

ASCs: adipose-derived stem cells
CM: conditioned medium
CC: co-culture
EMT: epithelial-to-mesenchymal transition
GM-CSF: granulocyte-macrophage colony-stimulating factor
GFP: green fluorescent protein
IL: interleukin
lnASCs: lean-derived ASCs
M-CSF: macrophage colony-stimulating factor
MCP-1: monocyte chemoattractant protein-1
obASCs: obese-derived ASCs
TAMs: tumor-associated macrophages
VEGF: vascular endothelial growth factor
VAT: visceral adipose tissue
WT: wild-type
