# Supplementary figures and images for: Survivin drives tumor-associated macrophage reprogramming: a novel mechanism with potential impact for obesity
Source: Cell Oncol (Dordr). 2021 Mar 12;44(4):777–92. doi: 10.1007/s13402-021-00597-x (PMC8338861; doi:10.1007/s13402-021-00597-x)

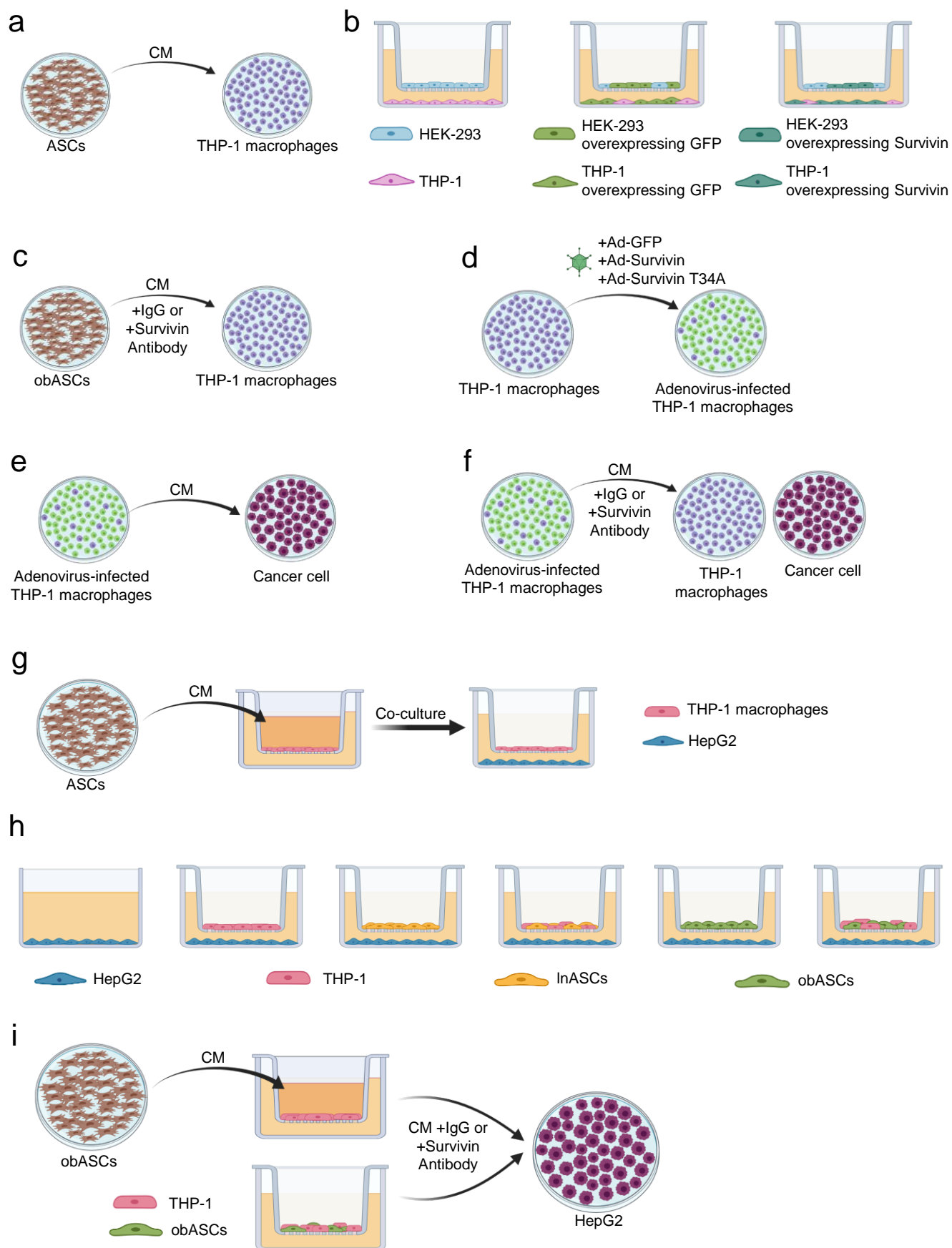

Supplementary Figure 1

Supplement: Supplementary file 1 — (PDF 356 kb) [file 13402_2021_597_MOESM1_ESM.pdf]
