# Supplementary material for: Survivin drives tumor-associated macrophage reprogramming: a novel mechanism with potential impact for obesity
Source: Cell Oncol (Dordr). 2021 Mar 12;44(4):777–92. doi: 10.1007/s13402-021-00597-x (PMC8338861; doi:10.1007/s13402-021-00597-x)
Supplement: Supplementary file 2 — (PDF 73 kb) [file 13402_2021_597_MOESM2_ESM.pdf]

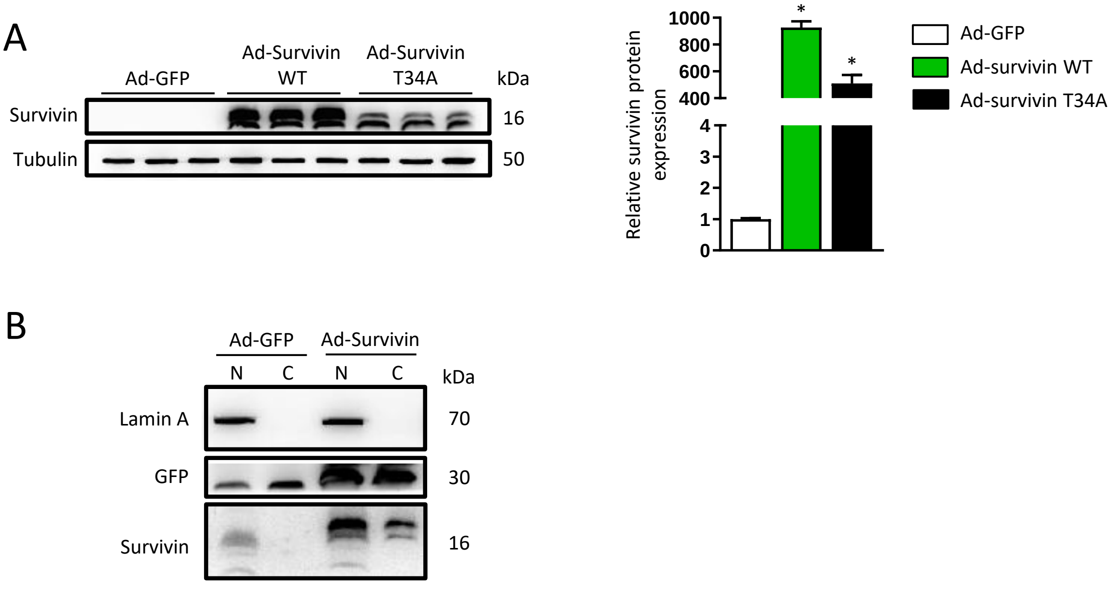


**Supplementary Figure 2. THP1-derived macrophages infected with a recombinant adenovirus expressing GFP (ad-GFP), wild-type survivin (WT) or mutated survivin (T34A).**

**A** Survivin protein expression. N=3: *p<0.05 *versus* ad-GFP. Tubulin was used as a loading control.

**B** GFP and survivin protein expression. Lamin A was used as nucleus marker.
