# Supplementary material for: Survivin drives tumor-associated macrophage reprogramming: a novel mechanism with potential impact for obesity
Source: Cell Oncol (Dordr). 2021 Mar 12;44(4):777–92. doi: 10.1007/s13402-021-00597-x (PMC8338861; doi:10.1007/s13402-021-00597-x)
Supplement: Supplementary file 3 — (DOC 46 kb) [file 13402_2021_597_MOESM3_ESM.doc]

**Supplementary Table 1. Human gene expression Taqman probes.** Results were calculated using the comparative Ct method and expressed relative to the expression of the housekeeping gene 18S.

| **Detector** | **Taqman probes** | **Gene probes** |
| --- | --- | --- |
| BIRC5 (survivin) | Hs04194392_s1 | Baculoviral IAP Repeat Containing 5 |
| Immune regulators | | |
| IL1b | Hs01555410_m1 | Interleukin 1 Beta |
| IL6 | Hs00985639_m1 | Interleukin 6 |
| TNF-α | Hs00174128_m1 | Tumor necrosis factor alpha |
| CCL2 | Hs00234140_m1 | C-C Motif Chemokine Ligand 2 |
| CCL3 | Hs00234142_m1 | C-C Motif Chemokine Ligand 3 |
| IL10 | Hs00961622_m1 | Interleukin 10 |
| PPARγ | Hs01115513_m1 | Peroxisome Proliferator Activated Receptor Gamma |
| KLF4 | Hs00358836_m1 | Kruppel-­like factor |
| Tumor associated macrophages membrane receptors | | |
| CD14 | Hs02621496_s1 | CD14 |
| CD68 | Hs02836816_g1 | CD68 |
| CD11b | Hs00167304_m1 | Integrin Subunit Alpha M |
| CX3CR1 | Hs01922583_s1 | C-X3-C Motif Chemokine Receptor 1 |
| CD45 | Hs04189704_m1 | Protein tyrosine phosphatase, receptor type, C |
| Invasiveness | | |
| MMP2 | Hs01548727_m1 | Matrix Metalloproteinase-2 |
| MMP9 | Hs00234579_m1 | Matrix Metalloproteinase-9 |
| TLR2 | Hs01872448_s1 | Toll Like Receptor 2 |
| TLR4 | Hs00152939_m1 | Toll Like Receptor 4 |
| VIM | Hs00958111_m1 | Vimentin |
| FN1 | Hs01549976_m1 | Fibronectin |
| CDH1 | Hs01023895_m1 | Cadherin 1 |
| ANGPTL4 | Hs01101123_g1 | Angiopoietin Like 4 |
| VEGFa | Hs00900054_m1 | Vascular Endothelial Growth Factor A |
| HIF-1α | Hs00153153_m1 | Hypoxia Inducible Factor 1 Subunit Alpha |
| TGF-β1 | Hs00998133_m1 | Transforming Growth Factor Beta 1 |
| EGF | Hs01099990_m1 | Epidermal Growth Factor |
| CASP1 | Hs00354836_m1 | Caspase 1 |
| Housekeeping | | |
| 18S rRNA | Hs03928985_g1 | 18S ribosomal RNA |
